# It’s What and When You Eat: An Overview of Transcriptional and Epigenetic Responses to Dietary Perturbations in Pancreatic Islets

**DOI:** 10.3389/fendo.2022.842603

**Published:** 2022-03-10

**Authors:** Matthew R. Brown, Aleksey V. Matveyenko

**Affiliations:** ^1^ Department of Physiology and Biomedical Engineering, Mayo Clinic College of Medicine and Science, Rochester, MN, United States; ^2^ Division of Endocrinology, Metabolism, Diabetes, and Nutrition, Department of Medicine, Mayo Clinic College of Medicine and Science, Rochester, MN, United States

**Keywords:** epigenetics, pancreatic islet, type 2 diabetes mellitus, high-fat diet, ketogenic diet, low-protein diet, intermittent fasting, time-restricted feeding

## Abstract

Our ever-changing modern environment is a significant contributor to the increased prevalence of many chronic diseases, and particularly, type 2 diabetes mellitus (T2DM). Although the modern era has ushered in numerous changes to our daily living conditions, changes in “what” and “when” we eat appear to disproportionately fuel the rise of T2DM. The pancreatic islet is a key biological controller of an organism’s glucose homeostasis and thus plays an outsized role to coordinate the response to environmental factors to preserve euglycemia through a delicate balance of endocrine outputs. Both successful and failed adaptation to dynamic environmental stimuli has been postulated to occur due to changes in the transcriptional and epigenetic regulation of pathways associated with islet secretory function and survival. Therefore, in this review we examined and evaluated the current evidence elucidating the key epigenetic mechanisms and transcriptional programs underlying the islet’s coordinated response to the interaction between the timing and the composition of dietary nutrients common to modern lifestyles. With the explosion of next generation sequencing, along with the development of novel informatic and –omic approaches, future work will continue to unravel the environmental-epigenetic relationship in islet biology with the goal of identifying transcriptional and epigenetic targets associated with islet perturbations in T2DM.

## Introduction

Type 2 diabetes mellitus (T2DM) is one of the major health challenges facing today’s society and projected to afflict nearly 1 in 3 people by year 2050 (1). T2DM is associated with a drastic increase in population morbidity/mortality, and more recently, has been shown to exacerbate adverse outcomes associated with COVID-19 (2). For these reasons, it is imperative to improve our understanding of the molecular mechanisms driving the increasing prevalence of T2DM which can lead to the development of novel therapeutic and preventative approaches. The pathophysiology of T2DM is mediated by complex interactions among diverse environmental and genetic susceptibilities (3), which ultimately culminate in the development of pancreatic islet failure characterized mainly by compromised β-cell insulin secretory function and loss of β-cell numbers. Although underlying genetics contribute to the pathogenesis of T2DM, environmental and epigenetic factors appear to be the primary drivers of this disease (4). Specifically, recent evidence suggests that modern changes in diet macronutrient composition (i.e., increased intake of saturated fats and refined sugars) along with disrupted circadian timing of food intake due to misalignment of circadian light/dark and fasting/feeding cycles has contributed to the induction of pancreatic islet failure and an overall increase in the predisposition to T2DM.

Indeed, pancreatic islets are constantly challenged by alterations in the timing and macronutrient composition of our diets to maintain adequate hormonal outputs for proper regulation of systemic glucose homeostasis. Specifically, the islet is required to integrate signals related to nutrient and energy status, circadian timing, and incoming neurohormones – all which can directly tune epigenetic, transcriptional, and physiological outputs. Glucose, for instance, directly suppresses (*via* increased ATP : AMP ratio) the AMP-activated protein kinase (AMPK) nutrient sensing pathway which controls transcriptional effectors critical to islet function such as the hepatocyte nuclear factor 4 alpha (HNF4α) (5, 6). During fasting and glucose restriction, AMPK-mediated phosphorylation/suppression of HNF4α prevents ectopic insulin secretion, in part, by thwarting transcriptional activation of the ATP-sensitive potassium channel, Kir6.2, required for depolarization-induced insulin secretion (7). Glucose-mediated activation of the mammalian target of rapamycin (mTOR) signaling pathway, meanwhile, initiates epigenetic/transcriptional programs that accelerate insulin processing and β-cell proliferation to meet the increased insulin demand (8). Hyperactivation of both mTOR and AMPK signaling can lead to β-cell failure highlighting the need for rhythmic nutrient cycles in order to maintain β-cell function (9, 10). Several metabolic intermediates such as acetyl-CoA and NAD^+^ are also co-factors required for histone acetylation and deacetylation, respectively, which in turn controls transcription factor recruitment and gene transcription (11). NAD^+^ mediated activation of the histone deacetylase sirtuin 1 (SIRT1), for example, is required in islets to transcriptionally inhibit uncoupling protein 2 (UCP2) *via* inactivation of the UCP2 promoter (12). UCP2 suppression is required to maintain GSIS given its role in uncoupling oxidative glucose metabolism (12), highlighting the tight link between metabolism, epigenetics, and islet function.

The circadian clock mediates the interaction between diet and the epigenome both at the organismal level through neurohormonal control over feeding behavior (13) and at the cellular level through direct transcriptional/epigenetic modulation (14). Molecularly, the circadian clock is encoded by a transcriptional-translation feedback loop (TTFL) which is driven by heterodimeric binding of the clock’s core transcription factors brain and muscle ARNT-like 1 (BMAL1) and circadian locomotor output cycles kaput (CLOCK) to palindromic E-box DNA binding motifs (14). Transcriptionally, BMAL1:CLOCK directly controls rhythmic patterns in gene expression of negative clock elements *(Per)* and cryptochrome (*Cry*), secondary transcription factors (i.e., D site of albumin promoter), and tissue specific functional effectors (15). Nutritional status and feeding time can shift transcriptional activation of this process *via* nutrient-stimulated signaling (16). For example, activation of insulin signaling in the liver during feeding has been shown to reset the circadian clock *via* mTOR-dependant Period activation, highlighting how the circadian clock is required to interpret and respond to incoming nutritional signals (17). Given this, genetic and environmental (i.e., shift work, inflammation) disruption of the β-cell circadian clock results in impaired GSIS and β-cell dysfunction due, in part, to impairments in rhythmic transcription of insulin secretion/exocytotic machinery (18–22). Outside of its direct transcriptional role, BMAL1 and CLOCK can also modify the epigenome both by acting as a pioneer transcription factor to recruit histone acetyltransferases (i.e., p300, CREB-binding protein), histone methyltransferases (i.e., mixed lineage leukemia 1), histone demethylase lysine-specific demethylase 1A (LSD1), and other transcription factors which cooperatively work together to promote gene activation (23) and by acting as histone acetyltransferase itself (CLOCK) (24). Taken together, the circadian clock acts a biological controller that integrates and responds to nutritional and behavioral input by precisely timing transcriptional/epigenetic programs to meet dynamic cellular demands.

Failed adaptation to this dynamic environment results in islet dysfunction due to impairments in these fundamental cellular processes (i.e., circadian rhythms, nutrient metabolism, mitochondrial function) which control the epigenome, undoubtably contributing to the development T2DM (25–28). Elucidating the physiological and molecular events that underly the islet’s response to various macronutrients and the overall timing of food intake has guided the chase for targeted T2DM therapies and novel preventative strategies; however, the progress has been limited because of the complex gene regulatory networks underlying these processes (29). The rapid development and adoption of genomic and epigenomic techniques are beginning to alter this narrative and are providing novel mechanistic insights into the regulatory mechanisms underlying the islet’s adaptation to various nutritional stimuli (30). As such, this review will examine the transcriptional and epigenetic mechanisms underlying the β-cell and islet response to the timing and composition of dietary macronutrients specifically overviewing current literature investigating islet adaptation to high-fat (HF) diets, high-sugar (HS) diets, ketogenic diets, low-protein diets, and time-restricted feeding/fasting-type diets (**Table 1**).

**Table 1:** Overview and comparison of the islet’s physiological, transcriptional, and epigenetic response to dietary interventions.

Dietary Interventions	Islet Phenotype	Transcriptional Adaptations	Epigenetic Adaptations	References
High fat diet/High fat + high sugar diet	Increased basal insulin and glucagon secretion Decreased β-cell and α-cell function Increased β-cell proliferation and mass Increased β-cell apoptosis	Rapid increase in cell cycle and proliferation transcripts Decreased β-cell identity, oxidative metabolism, insulin secretion, and exocytosis transcripts Increased immaturity (dedifferentiation), glycolytic, ER stress, and inflammatory transcripts Increased transcriptional entropy Similar transcriptional signature (by PCA) between HF and HFHS	Increased bivalency of Polycomb regulated promoters and enhancers Increased H3K27ac at loci regulating glycolytic and proliferative gene networks miRNA and lncRNA mediated increase in cell cycle and decrease of β-cell identity transcripts	(31–38)
High sugar diet	Increased basal glucagon secretion Reduced β-cell and α-cell function Reduced β-cell mass	Unknown	Unknown	(39–41)
Ketogenic diet	No change in basal insulin secretion No change in β-cell function No change in β-cell proliferation, mass, or apoptosis	Decreased cell cycle, ER stress, and inflammatory transcripts Increased insulin secretion and exocytosis transcripts Similar transcriptional signature (by PCA) between ketogenic and control islet	miRNA mediated decrease in cell cycle and increase in mitochondrial metabolism transcripts	(32, 42, 43)
Low protein/BCAA diet	Reduced basal insulin secretion No change in β-cell function	Unknown	Unknown	(44–46)
Time-restricted feeding	Reduced basal insulin secretion Increased circadian β-cell function No change in β-cell proliferation and mass	Increased protein/insulin processing, insulin secretion, kinase signalling and metabolic transcripts during feeding Increased lipid metabolism, inflammatory, and nutrient sensing transcripts during fasting Maintenance of core circadian clock transcripts and overall circadian rhythm in gene expression	Increased H3K27ac during feeding Maintenance of circadian rhythmicity in chromatin accessibility LSD1 recruitment and H3K4me1 demethylation of feeding activated loci Increased DBP activity at H3K27ac marked insulin secretory loci	(47, 48)
Alternate day fasting	Reduced basal insulin secretion Increased β-cell function Reduced β-cell apoptosis	Reduced p53 and IL-6 activity Increased p62 activity during fasting	Unknown	(49, 50)
Prolonged fasting	Increased β-cell proliferation and regeneration Reduced β-cell apoptosis	Increased β-cell identity and immaturity transcripts	Unknown	(51)

## Islet Functional and Epigenetic Response to High-Fat, High-Sugar Diets

The world’s current nutritional environment is dominated by inexpensive and easily accessible high saturated fat and high refined sugar/carbohydrate, energy dense, foods (52, 53). Both excess refined sugars (i.e., sucrose and fructose) and saturated fats have been implicated as causal factors in the development of obesity and T2DM; however, their relative contribution to pancreatic islet failure in T2DM is still under investigation (54–56). Despite differing perspectives on the dietary etiology of T2DM, long-term ingestion of a diet rich in saturated fatty acids and/or refined sugars has been clearly demonstrated to lead to increased visceral adiposity (57, 58), reduced energy expenditure (59, 60), peripheral insulin resistance (61, 62), and pancreatic islet dysfunction (39, 63–66). Enumerating the physiological, molecular, and genetic consequences of these dietary stressors on peripheral metabolic tissues may allow us to develop targeted therapies to reverse the effects of diet-induced metabolic dysfunction. While we will specifically focus on the islet’s response to HF and HS diets, please refer to Imamura et al. for an overview of the systemic effects of these diets (67).

The physiological mechanisms underlying islet adaptation to HF and HS diets have been thoroughly investigated over the last 40 years. It is important to note that the vast majority of rodent studies on this topic utilized only male mice partly due to the attenuation of deleterious metabolic effects of HF diets in female rodents (68). From rodents to canines and humans, prolonged HF feeding (typically containing 40-60% fat and ~20% carbohydrates) leads to a significant increase in basal insulin secretion (basal hyperinsulinemia) which demonstrates a positive correlation with saturated fatty acid intake and the extent of insulin resistance (69–72). Along with basal hyperinsulinemia, chronic HF diet has been also shown to reduce glucose-stimulated insulin secretion (GSIS) despite an absolute increase in insulin release and islet insulin content (31, 63, 64). The functional effects on GSIS with HF diet occur in parallel to adaptive increases in β-cell mass and β-cell proliferation potentially accounting for the noted increases in absolute insulin secretion and content in rodents (73, 74). Importantly, the observed decline in β-cell function and increase in β-cell mass with HF diet in rodents is not accelerated or significantly altered upon the replacement of carbohydrates with 20% sucrose in a typical HF, high sugar (HFHS) diet (32). HS diets (10-60% sucrose or fructose, 3-10% fat) have also been demonstrated to induce β-cell dysfunction due to impairments in first-phase GSIS (39–41) and increased β-cell apoptosis (39, 75). Other endocrine cell types of the islet, particularly the pancreatic α-cell, are also affected by long-term HF and HS diets as highlighted by the development of basal hyperglucagonemia and impaired glucose-induced suppression of glucagon secretion with both dietary interventions (31, 76, 77). As highlighted below, our understanding of the physiological, transcriptional, and epigenetic changes of the non β islet cells are currently lacking and additional studies will be needed to understand their contribution to the homeostatic response to dietary interventions associated with T2DM.

The effects of HF and HFHS diets on islet function and mass are associated with distinct changes in the islet’s transcriptional programs. At the initial onset of HF diet, the islet transcriptionally activates compensatory proliferative and protein biosynthesis pathways to increase overall insulin production and secretion (31). Indeed, within one-week of HF diet transcripts associated with cell proliferation are upregulated (e.g. *cyclin dependent kinase 1* [*Cdk1*]*, cell division cycle associated 2 [Cdca2], cyclin B1 [Ccnb1]*) to increase β-cell mass (33). This early transcriptional response requires both demethylation of E-box binding motifs and recruitment of MYC proto-oncogene (Myc), and likely other factors, to promoter/enhancer regions of genes regulating cell replication. Indeed, the circadian transcription factor BMAL1 which canonically binds to E-box motifs has also been demonstrated to be required for compensatory β-cell expansion in response to HF diet (19). Although there is an overall increase in β-cell number and insulin transcriptional levels with HF diet, the islets are characterized by an immature transcriptional signature highlighted by declines in identity factors such as neuronal differentiation 1 (*Neurod1*) and forkhead box A2 *(Foxa2)* along with impairments in glucose oxidation (i.e., glucokinase) and mitochondrial function (31). These are features also observed in failing, dedifferentiating β-cells in T2DM which results in decoupling of glucose intake from insulin secretion (25, 78). In fact, Dhawan et al. observed hypermethylation of the promoters of glucokinase (*Gck*) and urocortin 3 (*Ucn3*) with concurrent hypomethylation of glycolytic enzymes/dedifferentiation markers hexokinase 1 and 2 (*Hk1/2)* and lactate dehydrogenase (*Ldha)* in immature β-cells mirroring the early transcriptional changes with HF diet (79).

Continued long-term (> 10 weeks) HF-diet feeding eventually leads to islet transcriptional decompensation and eventual failure with enrichment for pathways regulating inflammatory and adaptive immune response, such as CXC chemokine receptor (CXCR) signaling, which has previously reported as a key feature of long-term *vs* short-term HF diet administration in other metabolic tissues due, in part, to increased immune cell infiltration and activation (31, 80). Importantly, activation of CXCR signaling has recently been demonstrated to facilitate β-cell functional failure and may contribute to β-cell demise with long-term HF diet ingestion (81). As previously noted, it is well documented that female rodents are protected from the long-term effects of HF feeding (82, 83). Consistent with this notion, transcriptional analysis of purified control and HF β-cells revealed that more than 50% of differential transcripts were sex dependant (82). HF-fed male β-cells were characterized by suppression of genes critical for oxidative phosphorylation and enrichment for islet amyloid polypeptide (*Iapp*), both features of failing β-cells in humans with T2DM (25, 65, 84). In contrast, female β-cells exhibited strong sex-specific differential expression of insulin synthesis and ER-stress related transcripts including activation of the endoplasmic reticulum oxidoreductase 1 beta (*Ero1b*) which is required for proinsulin processing and is directly correlated with the islet’s insulin secretory capacity (85). Lastly, the addition of dietary sucrose to a HF diet was found to not significantly alter the islet’s gene signature (by principal component analysis) and pathway activation/suppression relative to a control, low fat low sugar diet, likely underlying the similarities observed in islet function and mass between HF and HFHS diets (68). Although there are also likely to be similarities between the islet’s transcriptome in response to a HS diet versus HF diet, we currently lack any insight into this area and is an important avenue for future investigation.

Bulk islet analysis prevents identification of cell specific changes, including from β-cells, in response to different islet stressors such as diet. The recent advent of single cell technologies (86), endocrine sorting protocols (87), and fluorescent reporter models (88) have begun to address this challenge, providing a window into cell-specific transcriptome within the islet. Single-cell RNA-sequencing of short-term (1-week) HF fed islets identified 11 subpopulations of β-cells, along with populations of other islet cell types including α-cells (89). Amongst the captured β-cells, more than two-thirds (67%) of β-cells exhibited minimal differences in gene expression compared to their control counterparts, highlighting that the majority β-cells are initially ‘agnostic’ to HF diet – a feature lost with bulk analysis. This relative proportion of this agnostic β-cell group may contribute to the heterogeneous islet response to HF diet observed in rodents (90). The remaining β-cell populations exhibited downregulation of genes important for ER stress response with activation of inflammatory pathways, while minor populations exhibited activation of β-cell proliferative pathways and suppression of immune activation which likely drives the differential analyses observed in bulk findings (90). The α-cell transcriptional signature, previously unstudied in the context of HF feeding, was similarly resistant to transcriptional changes by HF diet with nearly half of captured α-cells not responding. Amongst the remaining ‘responders’, a major subpopulation of HF diet exposed α-cells had an immature islet transcriptional signature with increased *Neurogenin 3 (Ngn3)* and reduced *Ero1b* levels (91, 92). Although the functional significance and origin of this subpopulation remains unclear, it may precipitate the α-cell dysfunction and hyperglucagonemia typically observed with HF diet interventions (31, 76). As such, additional studies are urgently needed to reveal and target subpopulations of endocrine cell types driving metabolic dysfunction in response to HF-feeding.

Underlying the transcriptional dynamics associated with HF and HFHS feeding are highly active cis-regulatory elements (i.e., promoters, enhancers, repressors), a dynamic chromatin landscape, and single nucleotide polymorphisms (SNPs) which synergize to promote activation and repression of genetic networks (93). By integrating bulk and single-cell transcriptomics of β-cells from mice fed a HF diet with genome-wide islet chromatin states, investigators are beginning to elucidate how reprogramming of the epigenome contributes to HF diet-induced β-cell dysfunction (34). Using this approach, a subpopulation of dedifferentiated HF diet fed β-cells were determined to contain significantly more bivalent genetic loci compared to control β-cells specifically in Polycomb regulated domains. Bivalent regions are characterized by open regions of chromatin with both activator (histone H3 lysine 4 trimethylation; H3K4me3) and repressor (histone H3 lysine 27 trimethylation; H3K27me3) marks with low transcriptional activity indicated by the absence of RNA polymerase 2 signal in part due to Polycomb mediated silencing. This suggests that a population of HF diet-exposed β-cells have high transcriptional entropy which signals that a cell-type has lost its functional identity and may underly β-cell dedifferentiation process commonly associated with β-cell failure in T2DM (94, 95). Ectopic hyperacetylation of histone H3 lysine 27 (H3K27ac), a histone modification associated with active enhancer and promoter regions, within bivalent, Polycomb repressed loci has been proposed as a causal factor driving β-cell dedifferentiation (94). Supporting this notion, it was recently identified that bivalent promoter and enhancer regions of the β-cell dedifferentiation marker aldehyde dehydrogenase 1 member a3 (*Aldh1a3*) was found to be significantly hyperacetylated with HF feeding and directly correlated to its transcriptional ‘de-repression’ (35). Globally, HF diet fed islets exhibit several-fold more hyperacetylated loci compared to hypoacetylated loci. This may be rooted in impairments to the histone deacetylase SIRT1 which is attenuated with inflammation and lipotoxicity common to HF feeding (20, 96). Importantly, a majority of the hyperacetylated regions from the HF islets were in proximal active enhancer regions (<5kb from transcriptional start site [TSS]), particularly in promoter regions of glycolytic and proliferative genes permitting recruitment and activation of transcription factors and other transcriptional regulators required for β-cell expansion with HF diet (19, 33, 35, 36, 97, 98). The current evidence has begun to enumerate how HF feeding directly rewires the islet transcriptional and epigenetic environment toward an immature, dedifferentiated phenotype; however, future work is required to establish a direct link between epigenetic modifications and islet dedifferentiation/failure in T2DM.

A majority of single nucleotide polymorphisms (SNPs) lie in cis-regulatory regions and can significantly impact chromatin accessibility and architecture (99). In a series of studies, the Attie group aimed to enumerate expression quantitative trait loci (eQTL) which drive transcriptional, translational, and phenotypic response to a HFHS diet (45% fat, 34% sucrose) in an outbred mouse colony modeling human genetic diversity (100–102). By simultaneously capturing *in vivo* and *ex vivo* insulin secretory phenotypes, the islet’s transcriptome/proteome, and the 150,000 SNPs across ~500 outbred mice, the studies revealed several potential genetic drivers of HFHS induced β-cell dysfunction. They determined that several insulin secretory traits (basal insulin secretion, GSIS, insulin content) were highly driven by genetics involving several SNPs/loci which correlate with genome wide association study (GWAS) hits associated with T2DM in humans. For example, Keller et al. found that there was an eQTL hotspot at ~30 Mb on chromosome 1 associated with basal insulin secretion, among other secretory traits (100). This was mapped to protein tyrosine phosphatase non-receptor type 18 (*Ptpn18*) which exhibited a positive correlation between its expression and total insulin secretion. Genetic inactivation of *Ptpn18* prevented HFHS induced basal hyperinsulinemia leading to improved glucose tolerance and insulin sensitivity. These findings illustrate how the underlying genetic landscape directly controls both the transcriptional and phenotypic response of pancreatic islets due to diet-induced stress.

There is accumulating evidence that non-coding RNAs (ncRNA), including micro RNAs (miRNA) and long non-coding RNAs (lncRNA), play a critical role in the maintenance of islet function and identity (as reviewed by Eliasson and Esguerra (103)) through regulation of the islet’s transcriptional and epigenetic landscape. miRNAs are 20-24 nucleotide, single stranded RNAs that can modify gene expression through direct silencing and interference with post-transcriptional RNA molecules (104). Indeed, differential activation of the miRNA-ome has been demonstrated to be a contributing factor in the islet’s response to HF diet (37, 105). miR-802, the top-most upregulated miRNAs with HF diet, for instance was found to repress the β-cell identity transcription factor neuronal differentiation 1 (*Neurod1*) and was postulated to play a key role mediating HF-induced β-cell dedifferentiation (105). Several miRNAs have also been implicated as causal factors driving β-cell expansion with HF diet (37, 106–108). For example, Jacovetti et al. found that suppression of miR-338-3p is required for activation of survivin (*Birc5*) which is necessary for β-cell expansion due to its multifaceted role as an inhibitor of cell death and positive regulator of cell division (109). Similar to miRNAs, lncRNAs (>200 nucleotides) also have been found to modulate β-cell function and survival through control of epigenetic, transcription and post-transcriptional landscape (110). Mirroring the role of miRNAs in β-cell dedifferentiation due to HF diet, PDX1 Associated lncRNA (*PLUTO*), which is required for *Pdx1* activation (111) and 1*810019D21Rik* (*ROIT*) which is required for *NK6 Homeobox 1 (Nkx6.1)* transcriptional activation were identified as two of the top-most downregulated lncRNAs with HF feeding (38). LncRNAs such as XLOC_010971 (β long intergenic noncoding RNA 2, *βlinc2*) and XLOC_013310 (β long intergenic noncoding RNA 3, *βlinc3*), have also been identified as potential mediators of β-cell decompensation with HF feeding. Respective gain- and loss-of-function studies of *βlinc2* and *βlinc3* highlighted previously unrecognized role of these lncRNAs in the negative regulation of β-cell survival *via* activation of nuclear factor-κB’s (NF-κB) nuclear translocation, a key step in β-cell apoptosis due to diabetogenic stress (112). This suggests that HF-induced changes to lncRNAs expression may contribute to β-cell demise and decompensation with long-term HF feeding. In summary, the islet’s lncRNA landscape is significantly perturbed with diet-induced metabolic dysfunction which can potentially modulate adaptation in both pancreatic β-cell function and mass.

## Islet Response to the Low-Carbohydrate, High-Fat Ketogenic Diet

The ketogenic diet, consisting of ~70-80% fat, 1-10% carbohydrates and 10-20% protein, has recently re-emerged as a promising dietary therapy for T2DM nearly 100 years since it was first coined by Mayo Clinic physician Dr. Russel Wilder in 1921 as a therapy for epilepsy and later diabetes (113, 114). Modern clinical studies for the most part have reproduced the metabolic benefits of inducing a nutritional state of ketosis by demonstrating that a ketogenic diet effectively reduces body weight, lowers glycemia, and attenuates hyperinsulinemia in obese, T2DM subjects (115–117). For instance, Michalczyk et al. found that hyperinsulinemic, obese women who followed a ketogenic diet for 12-weeks exhibited a 54% reduction in fasting insulin with a 20% lowering of fasting glucose relative to baseline (117). Spurred by this clinical phenotype, several groups have investigated the islet’s physiological and genetic adaptation to a ketogenic diet in both basal and diabetic states (32, 118–121). Compared to a control low-fat, high-carbohydrate diet, the ketogenic diet induced glucose intolerance due to prevailing hypoinsulinemia and significantly reduced β-cell (and α-cell) mass (118, 119, 122). When compared to a traditional HF or HFHS diet; however, Her et al. demonstrated that 12-weeks of ketogenic diet attenuated hyperinsulinemia, enhanced β-cell secretory function, and in turn restored euglycemia (32). Similar effects have been observed for other models of diabetes including the leptin deficient ob/ob model (120), the Akita model of impaired insulin folding (121), and even the type 1 diabetes, streptozotocin (STZ) model (123).

Based on the noted effects of a ketogenic diet on islet function and mass, Her et al. also compared the islet’s transcriptional signature in response to a ketogenic diet relative to HF and HFHS diets (32). Using PCA analysis, the transcriptional signature of ketogenic islets was found to be more closely related to control islets compared to HF or HFHS islets with only 15% of genes were commonly regulated between ketogenic and HF/HFHS diets. Moreover, gene-set enrichment analysis revealed that ketogenic feeding significantly attenuated transcripts associated with cell division and activated genes regulating insulin secretion, relative to HF and HFHS diet islets mirroring the phenotypic features of the ketogenic islet. Ketogenic islets were also characterized by significant reduction in the expression of genes critical for immune activation and ER stress relative to control, low-fat diet highlighting its potential role in protecting the islet from chronic inflammation and the unfolded protein response common to obesity and T2DM (26, 124). In line with these findings, Tattikota et al. found that 4-5 weeks of ketogenic diet attenuated β-cell proliferation in ob/ob islets, in part, due to restoration (20-fold) of miR-184 expression, which is a potent inhibitor of cellular proliferation by disrupting Myc signaling (42, 125). miRNAs have also been suggested to play a key role in enhanced mitochondrial metabolism with ketogenic diet, a key step in GSIS which is dysfunctional with HF diet (43, 126). Specifically, miR-125b was shown to be significantly downregulated with ketogenic diet and was demonstrated to directly target mitochondrial fission process 1 (Mtfp1), among other mitochondrial and lysosomal transcripts. Inhibition of Mtfp1 has been shown to result in mitochondrial fragmentation, a phenotypic trait of the T2DM islet (127), and therefore suppression of miR-125b may contribute to the maintenance of glucose oxidation and insulin secretion observed with ketogenic diets (128). Despite limited insights into the epigenetic effects of ketogenic diets on the islet, these initial studies have begun to provide potential mechanistic insights underlying the therapeutic benefits of ketogenic diet in T2DM.

## Islet Response to Protein- and Branched-Chain Amino Acid (BCAA) Restricted Diets

Amino acids play a multifactorial role in maintenance of islet survival and function by acting as building blocks for islet machinery, serving as signaling molecules for nutrient sensing pathways, and by directly inducing nutrient-stimulated insulin and glucagon secretion (129). As such, dietary protein and amino acid intake can tremendously impact both the function and development of the endocrine pancreas, depending on the cellular need for amino acids. Specifically, protein restriction during gestation and maturation can have long-term detrimental effects on the islet (130–132). In contrast with the need for dietary protein in normal β-cell development and maturation, an over-supply of protein and amino acids, particularly branched-chain amino acids (BCAA) leucine, isoleucine, and valine, is associated with the development of T2DM (133). In turn, diets with reduced BCAA content have been demonstrated to restore euglycemia mainly through improvements in insulin sensitivity in both obese/diabetic rodents and humans (44, 45). This is accompanied by a reduction in β-cell insulin hypersecretion with Karusheva et al. reporting a 30% decrease in post-prandial insulin secretion in T2DM subjects following 4-weeks of a diet lacking BCAA (134). Consistently, it was found that rodents fed a BCAA and amino acid (AA) low diet for 3-weeks had reduced overall insulin secretion due to a decrease in β-cell metabolic flux and corresponding increased glucose-stimulated Ca^2+^ oscillatory efficiency (44). More recently, Cummings et al. extended this work and observed a ~2-fold decrease in plasma insulin 5-weeks following a switch from a HFHS diet to either a BCAA or AA deficient diet which was associated with reduced β-cell mitochondrial membrane potential (a marker of basal hyperinsulinemia) (45, 46). Although the islet’s genetic and epigenetic adaptations to a BCAA/AA low diet remain largely unexplored, these phenotypic studies suggest the need to understand the molecular mechanisms underlying improvements in islet function associated with BCAA/AA-restricted diets.

## Islet Response to Time-Dependent Regulation of Food Intake

Periodic fasting and, in turn, fasting mimicking diets have gained rapid popularity as potential therapeutic strategies for a wide array of metabolic and degenerative diseases. In contrast with absolute caloric restriction (CR) which limits daily nutritional energy by as much as 50% without altering meal timing or frequency, periodic fasting-type diets consists of extended periods ranging from 12 h to weeks without any caloric intake with subsequent ‘caloric-blind’ feeding periods lasting as little as a few hours (135). Although caloric restriction has been demonstrated as a therapeutically beneficial approach to restore islet function in both diabetic mice and humans (136–138), adherence to prolonged periods of caloric restriction has been challenging in humans (139). In support of fasting-type diets, Gil and Panda demonstrated that erratic and extended feeding patterns in humans significantly contribute to obesity which when normalized with prolonged fasting resulted in reduced body weight and improved health (140). Fasting can be divided into two distinct subtypes: intermittent fasting (IF) and periodic fasting (PF). IF is defined as 12 h to 2 days of fasting followed by *ad libitum* feeding periods that last between a few hours and 5 days. As such, IF is comprised of several fasting regimens such as alternative day fasting (ADF) (24 h fasting: 24 h feeding), 5:2 diet (2 days fasting: 5 days feeding), and time-restricted feeding (tRF) (12 to 18 h fasting: 6 to 12 h feeding). Meanwhile, PF is defined by 2 or more days of fasting, lasting up to several weeks, with long ad libitum refeeding intervals spanning at least 1-week. PF also encompasses fasting mimicking diets (FMD) which are made up of ~10-50% typical calories and consist of high fat (>50%) with low protein (<10%) and carbohydrate (<40%) content. In line with isocaloric PF, FMD last 2 or more days and are interspersed with at least 7 days of ad libitum feeding. The therapeutic interest in IF and PF is not an entirely new concept with Otto Folin and W. Denis demonstrating in 1915 that prolonged fasting ameliorated obesity and metabolic syndrome (141). Although recent studies have re-emphasized the therapeutic benefits of fasting, the molecular mechanisms underlying beneficial effects of these diets on the islet are only starting to be elucidated more than 100 years later.

While IF and PF have been clearly shown to ameliorate metabolic dysfunction across several tissues (i.e., liver, adipose, brain), we will focus specifically on the pancreatic islet’s physiological and, in turn, epigenetic response to fasting. For a comprehensive overview of the effects of IF and PF on whole organism physiology, please refer to Longo et al. (142). Prolonged fasting-type diets have been demonstrated to enhance β-cell health, primarily by limiting chronic insulin demand during metabolic stress. The benefits of acutely reducing insulin secretion on β-cell health and function for T2DM gained prominence in the 1970s when Greenwood, Mahler, and Hales suggested that β-cell overstimulation leads to impaired GSIS when they found that diazoxide treatment, a potent inhibitor of insulin secretion, restored β-cell function in T2DM subjects (143). This was followed by a series of studies demonstrating that “β-cell rest” or acute inhibition of insulin secretion, could restore β-cell function (first phase insulin secretion), insulin/Ca^2+^ pulsatility, and insulin content in obesity and T2DM (144–148). Chronic β-cell stimulation common to high fat feeding/obesity (149) and circadian disruption (21, 22) result in both β-cell dysfunction and apoptosis which have been shown to be reversed by ADF (49, 150), tRF (47, 151, 152), and a FMD (153). Specifically, a 12-week ADF regimen protected mice from HF diet induced β-cell dysfunction and failure (49), which was associated with enhanced GSIS, restored islet insulin content, and reduced apoptosis contingent on ADF activation of the autophagy-lysosome pathway. Although fewer studies have examined the physiological effects of PF on islet function, Kolakowski et al. observed in 1970 that 7-day PF followed by 3-days of tRF resulted in a 2.5-times increase in insulin secretory capacity (154). PF *via* FMD has also been demonstrated to modulate functional β-cell mass in insulin-deficient and HF diet models of diabetes (51, 153); however, the ability to translate these findings are confounded by the limited replicative capacity of human β-cells (155). Taken together, the physiological characterization of fasting-type diets has demonstrated their ability to promote β-cell rest, restore islet function, and potentially regenerate β-cell mass, highlighting the importance of cyclic fasting-feeding to maintain β-cell health.

Underlying the noted physiological effects of IF and PF on pancreatic islet function and survival in T2DM are dramatic changes to the islet’s transcriptional and epigenetic identity. Under control conditions, islets isolated from C57B/6J mice following 6-days of tRF exhibit clear transcriptional and epigenetic differences during periods of feeding versus fasting (48). Specifically, fasted islets exhibit enrichment for genes associated with lipid/fatty acid/carbohydrate metabolism and nutrient sensing (Forkhead box O [FoxO] and mTOR signaling). Indeed, FoxO signaling is activated in the β-cell upon glucose and nutrient restriction and is required for maintaining diurnal metabolic flexibility between carbohydrates and lipids (156, 157). In contrast, fed islets are significantly enriched for genes associated with protein processing/export, kinase signaling, and amino acid metabolism, all which are required for GSIS. This is consistent with circadian analysis of the mouse islet’s transcriptome which similarly found significant enrichment for protein processing/export, insulin secretion and amino acid metabolism during the animals active, feeding period and corresponding activation of nutrient sensing (mTOR), inflammatory signaling (tumor necrosis factor, cytokine receptor, nuclear factor kappa B [NFκB]) and fatty acid metabolism during inactive, fasting periods suggesting that rhythmic feeding/fasting cycles drive the islet’s transcriptional identity (158). In line with the noted changes in gene expression, Wortham et al. (48) found that nearly half of all active enhancers and promoters (~19,000 by H3K27ac) were differentially acetylated (active) during fasting *vs*. feeding, with 80% hyperacetylated with feeding. Although not directly compared, the hyperacetylation exhibited in the HF diet islet may be partially driven by disruption in the circadian feed-fast cycle which occur with HF feeding (159). The leptin receptor deficiency model of T2DM (Lepr^db/db^), which also exhibits impaired circadian food intake rhythms (160), similarly exhibits increased H3K27ac and histone H3 lysine 4 monomethylation (H3K4me1; activating chromatin mark which co-occurs with H3K27ac) signal in regions associated with feeding but not in regions associated with fasting. Importantly, regions hyperacetylated are associated with LSD1 binding where it acts as a brake on transcription by demethylating H3K4, likely contributing to β-cell rest (161). Consistently, β-cell specific LSD1 deletion physiologically and epigenetically phenocopies the HF diet state: basal hyperinsulinemia, impaired GSIS and activation of a ‘stressed/immature’ gene signature (i.e., *hexokinase1 [Hk1], 6-phosphofructo-2-kinase/fructose-2,6-biphosphatase 3 [Pfkfb3], jun proto-oncogene [Jun], fos proto-oncogene [Fos], nuclear factor, interleukin 3 regulated [Nfil3]*). Future studies will likely be needed to unravel whether mistimed feeding, nutrient overabundance, or both are factors driving hyperacetylation and β-cell dedifferentiation with HF diet and T2DM.

Circadian disruption and shift work are environmental stressors characteristic of impaired feeding/fasting rhythms and have been directly linked to the development of islet failure in T2DM (22, 162). Pancreatic islets isolated from circadian disrupted (*via* constant light) mice exhibit complete ablation of circadian rhythms in both gene expression and chromatin accessibility (47). Providing further evidence that rhythmic feeding/fasting cycles drive islet transcription, tRF was shown to rescue transcriptional and epigenetic (chromatin accessibility) rhythms, despite global circadian disruption, of genes/loci annotated to pathways regulating circadian rhythms along with β-cell function (i.e., insulin secretion and exocytosis) and rest pathways (i.e., FoxO signaling, fatty acid metabolism) active during feeding and fasting, respectively. The interaction of nutrient sensing pathways with the circadian clock has been shown to be a key regulator underlying feeding/fasting driven rhythms in transcription and chromatin accessibility. Under circadian disruption conditions, where feeding cycles are disrupted, the circadian transcription factor D site of albumin promoter (Dbp) is highly suppressed (~20-fold at peak). During extended fasting periods with tRF, Dbp is activated by FoxO1 (163), and likely other factors (164), where it begins to bind active promoter and enhancer regions (~75% overlap with H3K27ac) of genes associated with insulin processing and secretion to prepare the β-cell (i.e., β-cell rest) for subsequent feeding periods. Histone modifying factors such as LSD1 likely also play a role in this process and have been shown to be required for circadian transcription factor activation in the liver due to diurnal differences in nutrient availability and could play a similar role in the islet; however, future studies will be required to establish this direct link (165).

Fasting type diets can also directly modulate transcriptional mechanisms underlying β-cell proliferation and survival. Indeed, ADF impeded HF diet induced β-cell apoptosis due in part to suppression of interleukin-6 and tumor protein p53, two key regulators of DNA damage response, and activation of autophagic/lysosomal flux (49, 50). Given that PF has been found to be preserve β-cell mass in several models of β-cell loss, it seems likely that extended fasting protects the β-cell from p53-mediated DNA damage associated with diabetogenic stress (51, 166). Subsequent refeeding following a prolonged fasting period results in a mixed islet transcriptional phenotype: upregulation of β-cell identity genes such as *MAF bZIP transcription factor A (Mafa), pancreatic and duodenal homeobox 1 (Pdx1), insulin 2 (Ins2)*, and *glucose transporter 2 (Glut2)* with a parallel induction of pluripotency/immaturity markers *Nanog homeobox (Nanog), DNA methyltransferase 3 beta (Dnmt3b), SRY-Box transcription factor 17 (Sox17), Neurogenin 3 (Ngn3)* and *GATA-binding factor 6 (Gata6)* (51). Chronic activation of these pluripotency and secretory factors due to constant feeding, common to both obesity and circadian disruption (22, 140), may play a role in precipitating β-cell dedifferentiation and dysfunction in T2DM. Together, this provides further evidence supporting the importance of extended fasting periods to maintain β-cell health; however, future transcriptional and epigenetic studies are still needed to fully characterize islet cell specific changes in response to modified feeding/fasting cycles which may underly β-cell failure in T2DM.

## Conclusion

The interaction between the pancreatic islet and dietary inputs has long been appreciated as a key piece in the etiology of islet failure in T2DM. More recent studies suggest that the temporal regulation of food intake is another critical regulator of islet function, and when perturbed, contributes to the development of T2DM. Indeed, lifestyle-driven changes in diet composition and daily patterns of food intake appear to be primary environmental factors contributing to ever-increasing rise in the incidence of the metabolic diseases worldwide. This review outlined the current literature highlighting the complexity in gene regulatory networks dictating the islet’s response to changes in the composition of dietary macronutrients, specifically proteins, carbohydrates, and fatty acids, and the timing of their consumption (i.e., intermittent fasting, time-restricted feeding) throughout life. The current evidence underlying successful versus failed islet adaptation to dietary interventions points to the importance of the minimalist motto: “less is more”. Less basal insulin secretion, less proliferation, less transcriptional entropy, less bivalent promoters and enhancers, less acetylation of H3K27, and less feeding time are all common threads associated with maintaining robust meal-stimulated insulin secretion and mature beta cell phenotype and genotype. A diet and lifestyle that allows the β-cell and likely other islet cell types time to “rest and repair” mediated by proper alignment of fasting/feeding cycles with internal circadian clocks appears to be a critical factor in preventing β-cell hyperactivation and eventual demise. With the ongoing development of additional low-input and single-cell epigenetic techniques (i.e., CUT&RUN, SHARE-seq) (167, 168) and further adoption of current technologies (i.e., RNA-seq, ATAC-seq, ChIP-seq), future studies will likely provide additional insights into causal transcriptional and epigenetic mechanisms underlying how each islet cell type responds to the timing and composition of their diet, their contribution to T2DM development, and their potential as a therapy to ameliorate islet dysfunction in T2DM.

## Author Contributions

MB performed the systematic literature search and preparation of the manuscript. AM oversaw the literature review and wrote the manuscript with MB. All authors contributed to the article and approved the submitted version.

## Conflict of Interest

The authors declare that the research was conducted in the absence of any commercial or financial relationships that could be construed as a potential conflict of interest.

## Publisher’s Note

All claims expressed in this article are solely those of the authors and do not necessarily represent those of their affiliated organizations, or those of the publisher, the editors and the reviewers. Any product that may be evaluated in this article, or claim that may be made by its manufacturer, is not guaranteed or endorsed by the publisher.
